# Long-term follow-up of a Tay-Sachs disease patient with cherry-red spot

**DOI:** 10.1016/j.ajoc.2026.102548

**Published:** 2026-02-20

**Authors:** Noriko Tsutsumi, Sonoko Sakata, Riyu Ikari, Nobuyuki Ebihara

**Affiliations:** a Department of Ophthalmology, Juntendo University Urayasu Hospital, 2-1-1 Tomioka, Urayasu-shi, Chiba, 279-0021, Japan; b Department of Ophthalmology, Inoue Eye Hospital, 4-3 Kandasurugadai, Chiyoda-ku, Tokyo, 101-0062, Japan

**Keywords:** Cherry-red spot, Tay-Sachs disease, Sphingolipidoses, Gangliosidosis, Hexosaminidase A, Lysosomal storage disorders, Macular degeneration

## Abstract

**Purpose:**

To describe the clinical progression and ophthalmic findings in a Japanese boy with Tay-Sachs disease at ages 5 and 8 months.

**Results:**

The patient was born at 38 weeks of gestation and developed normally until motor skill delays were identified at age 1 year and 1 month. At that time, brain magnetic resonance imaging revealed diffuse T2 hyperintensity in the bilateral basal ganglia. At age 1 year and 6 months, he was able to fix and follow objects, and the optic discs appeared normal, but bilateral cherry-red spots were observed in the maculae. Based on these findings, together with hypersensitivity to sound and markedly reduced β-hexosaminidase A activity, Tay-Sachs disease was biochemically diagnosed. By age 2 years and 1 month, the patient had lost the ability to fixate on and follow objects. Fundoscopy at age 4 years and 4 months revealed optic atrophy and reduced cherry-red spots, electroretinography (ERG) at age 4 years and 9 months showed a complete loss of retinal responses, and optical coherence tomography at age 5 years and 3 months demonstrated retinal thinning. Long-term observation revealed progressive degeneration, consistent with previous reports. Lipid deposition in all retinal layers was considered to contribute to retinal atrophy.

**Conclusions:**

This report highlights progressive retinal degeneration with optic atrophy, retinal thinning, and reduction in cherry-red spots. The flat ERG waveform in this patient suggests more advanced retinal involvement than in previous reports and provides insights into the ocular manifestations of Tay-Sachs disease.

## 1 Introduction

Tay-Sachs disease is a rare, progressive, autosomal recessive neurodegenerative disorder caused by a deficiency of the enzyme β-hexosaminidase A, which leads to the accumulation of GM2 gangliosides, particularly in the retina, brain, and nervous system.^1^ Clinical presentations are categorized into infantile, juvenile, and adult-onset forms, with the infantile form being the most severe. Symptoms typically emerge at age 6 to 7 months and include developmental delays, muscle weakness, hypersensitivity to sound, and the hallmark cherry-red spot observed on fundus examination. No effective treatments have been established, but gene therapy with adeno-associated virus vectors is under investigation.^2^ The characteristic cherry-red spot results from retinal opacities due to abnormal lipid accumulation in retinal ganglion cells. These opacities are most prominent around the central fossa, where ganglion cell density is highest; in contrast, the foveal and peripheral regions, where ganglion cell density is lower, are relatively unaffected.^3^

## 2 Case report

The patient was a Japanese boy born at 38 weeks of gestation with a birth weight of 3310 g. Pregnancy and delivery were uneventful. He had no notable medical or family history, and no developmental delays were observed during the neonatal period. However, at age 1 year and 1 month, delays in motor development were identified, followed by a regressive course with progressive loss of previously acquired skills. Brain magnetic resonance imaging revealed diffuse T2-weighted hyperintensity in the bilateral basal ganglia. Cerebrospinal fluid analysis showed normal lactate and pyruvate levels, but magnetic resonance spectroscopy demonstrated a lactate peak, initially suggesting Leigh syndrome; however, mitochondrial gene testing was negative. There was no hepatomegaly, and abdominal examination revealed no abnormalities. During the course, the patient exhibited startle responses to auditory stimuli. At age 1 year and 6 months, he was referred to our department for a comprehensive systemic evaluation.

**Fig. 1:**
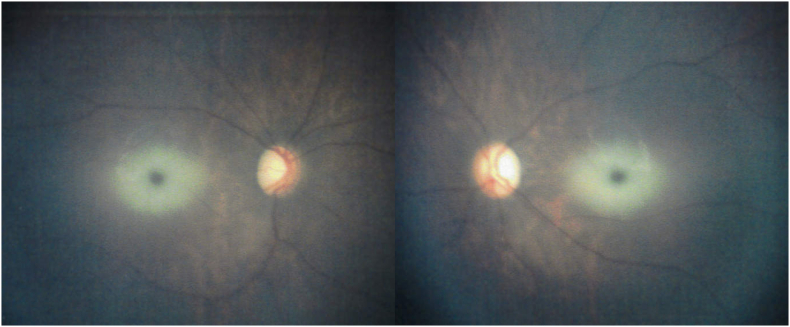
Fundus photograph at age 1 year and 6 months showing a cherry-red spot. (For interpretation of the references to colour in this figure legend, the reader is referred to the Web version of this article.)

**Fig. 2:**
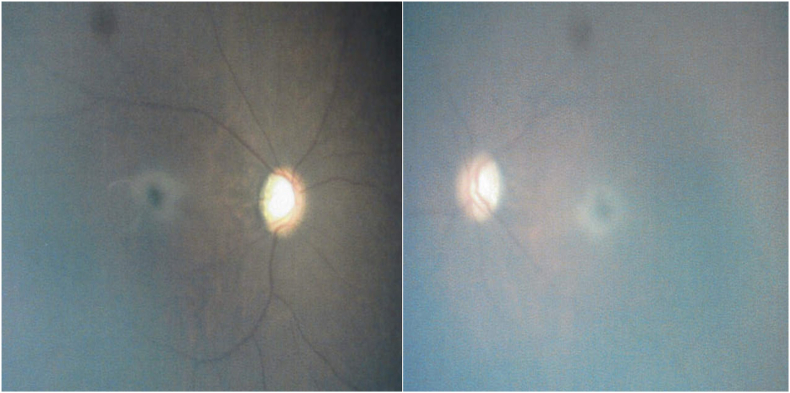
Fundus photograph taken at 4 years and 4 months of age showing a fading cherry-red spot and progressive optic atrophy. (For interpretation of the references to colour in this figure legend, the reader is referred to the Web version of this article.)

**Fig. 3:**
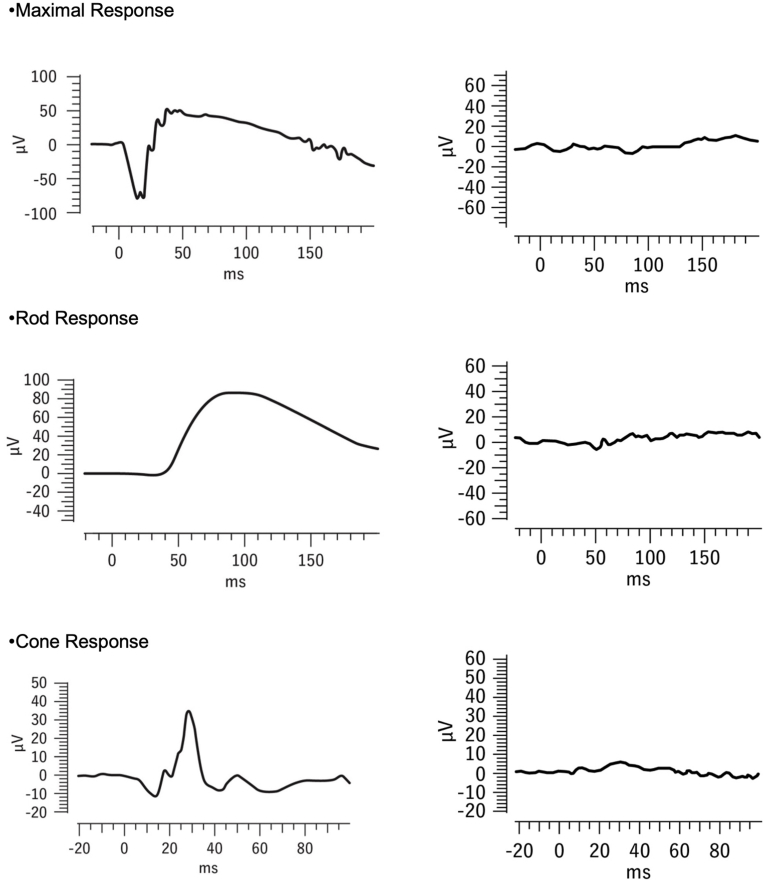
ERG recordings obtained using skin electrodes according to the ISCEV standard. Representative ERG traces from a normal individual are shown on the left, and those from the patient at age 4 years and 9 months are shown on the right. ERG responses were extinguished in both eyes.

**Fig. 4:**
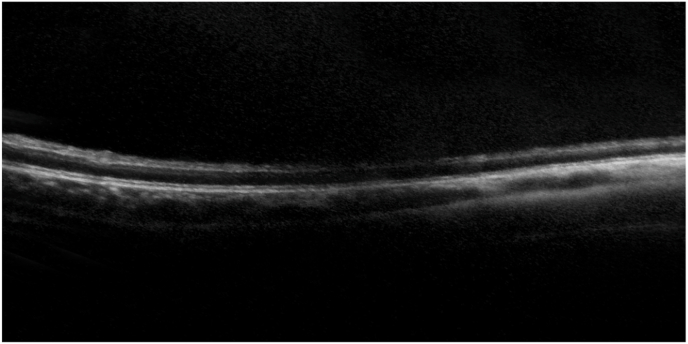
OCT scans at age 5 years and 3 months of a patient with Tay-Sachs disease, showing generalized retinal layer thinning with hyporeflectivity in the foveal and parafoveal regions and hyperreflectivity in the peripheral inner layer.

At the time of referral, the patient was able to fix and follow objects, and the optic discs appeared normal; however, bilateral cherry-red spots were observed in the maculae (Fig. 1). Based on these findings and hypersensitivity to sound, Tay-Sachs disease was suspected. Enzyme assay of skin fibroblasts demonstrated a marked deficiency of β-hexosaminidase A activity, with total β-hexosaminidase activity preserved, consistent with a biochemical diagnosis of Tay-Sachs disease. By age 2 years and 1 month, the patient was no longer able to fixate on or follow objects. Fundoscopy at age 4 years and 4 months revealed completely pale and atrophic optic discs and a reduction in the retinal opacification surrounding the cherry-red spots (Fig. 2). At age 4 years and 9 months, electroretinography (ERG) performed with skin electrodes and the RETeval® system, according to the International Society for Clinical Electrophysiology of Vision (ISCEV) standard, demonstrated extinguished responses in both eyes (Fig. 3), indicating a complete loss of maximal, rod, and cone responses. Representative ERG recordings are shown in Fig. 3. At age 5 years and 3 months, handheld optical coherence tomography (OCT) revealed diffuse retinal atrophy with marked thinning of the full-thickness retina (Fig. 4). Hyporeflectivity was observed throughout the inner retinal layers in the foveal and parafoveal regions, whereas the peripheral retina exhibited hyperreflectivity of the inner retinal layers. At the time of writing, the boy was aged 5 years and 8 months and remained under observation.

## 3 Discussion

### 3.1 Long-term observation

The present case report focused on the long-term evolution of the cherry-red spot and associated retinal changes in Tay-Sachs disease. To our knowledge, few reports have describing both OCT and ERG findings in the advanced stage of Tay-Sachs disease. Previous studies have primarily described early infantile cases, whereas our case provides detailed documentation of structural and functional retinal changes observed during long-term follow-up into later childhood.

The infantile form typically manifests in early infancy with rapid neurodegeneration and an almost universal cherry-red spot, whereas the juvenile form shows slower progression and rarely presents with this finding. These distinctions indicate that the present case represents the classical infantile type.^4^ This patient was followed for a relatively long time, from age 1 year and 1 month to 5 years and 8 months. In a previously reported case, light perception was absent at age 1 year and 1 month, and cherry-red spots were observed in both eyes; the spots had diminished slightly at age 3 years and 4 months and continued to diminish until age 5 years and 8 months.^5^ Similarly, in our patient, the cherry-red spots diminished after visual responses declined.

In another previously reported case, ERG and visual evoked potential (VEP) testing were performed concurrently; the ERG results were nearly normal, but no VEP was observed.^6^ The visual transmission pathway from the optic nerve to the visual cortex has been suggested to degenerate before the retina,^7^ consistent with the course of the present case. Two other reports described nearly normal ERG results in patients aged 1 year and 2 months and 2 years and 2 months.^6^^,^^7^ To our knowledge, no other cases have been reported in which ERG responses have extinguished.

In our patient, ERG was performed at age 4 years and 9 months, older than in previous reports. In an autopsy study of Tay-Sachs disease patients, Gartner and Bronstein found that destruction of ganglion cells and near-complete atrophy of the ganglion cell layer had occurred in patients aged 1 year and 7 months and 1 year and 10 months, compared with those younger than 1 year and 1 month.^8^ Similarly, in the present case, retinal degeneration progressed throughout the observation period. With disease progression, loss of ganglion cells and inner-retinal degeneration contribute to diffuse retinal atrophy and fading of the cherry-red spots, consistent with the clinical course observed in our patient.^9^

### 3.2 Lipid deposition in the retina

Among diseases presenting with a cherry-red spot, conditions such as Niemann-Pick disease and GM1 gangliosidosis were considered.^9^ However, the presence of hypersensitivity to sound and markedly reduced β-hexosaminidase A activity led to the biochemical diagnosis of Tay-Sachs disease.

In Tay-Sachs disease, GM2 gangliosides primarily accumulate in retinal ganglion cells, producing perifoveal retinal opacification and the classic cherry-red spot. In GM2 gangliosidoses, lipid accumulation has been reported not only in retinal ganglion cells but also across all retinal layers,^10^^,^^11^ with deposits in amacrine cells and Müller cell processes indicating involvement of the entire inner retina.^11^^,^^12^ Similar changes have been reported in other lipid storage diseases, such as Niemann-Pick disease and neuronal ceroid lipofuscinosis.^13^^,^^14^ Like Tay-Sachs disease, these lysosomal storage diseases cause lipid accumulation in retinal ganglion cells. Weleber^15^ reported that some patients with neuronal ceroid lipofuscinosis show ERG findings similar to those observed in the present case.

Although the pathological basis of the OCT findings in our patient cannot be confirmed, we speculate that lipid deposition occurred throughout all retinal layers, resulting in atrophy of the entire retina and gradual fading of the cherry-red spot. With disease progression, inner-retinal degeneration leads to diffuse retinal atrophy and fading of the cherry-red spot, which, in advanced stages, correlates with extinguished ERG responses. There are no previous reports describing extinguished ERG responses in advanced Tay-Sachs disease. However, in neuronal ceroid lipofuscinosis, ERG responses are known to disappear in the advanced stage.^15^ Our case therefore provides valuable documentation linking retinal degeneration with ERG extinction in Tay-Sachs disease.

This study is limited by being a single case report; however, such rare cases remain valuable for understanding the ocular features of this disease. Another limitation is that genetic testing was directed toward mitochondrial disorders.

## 4 Conclusion

This case report highlights the clinical progression and ocular manifestations of infantile Tay-Sachs disease observed over an extended period. In particular, it emphasizes the gradual diminishment of the hallmark cherry-red spots, which correlated with progressive degeneration of the visual transmission pathway and worsening retinal atrophy. The unique finding of an extinguished ERG response at age 4 years and 9 months indicates advanced retinal degeneration that may extend beyond the ganglion cells to involve all retinal layers. These findings provide valuable insight into the natural history of Tay-Sachs disease and underscore the importance of long-term ophthalmologic monitoring in lysosomal storage disorders. Further studies are needed to elucidate the pathological mechanisms underlying full-thickness retinal atrophy and their implications for future therapeutic interventions.

## CRediT authorship contribution statement

**Noriko Tsutsumi:** Writing – review & editing, Writing – original draft. **Sonoko Sakata:** Investigation. **Riyu Ikari:** Investigation. **Nobuyuki Ebihara:** Supervision.

## Patient consent

Written consent to publish this case has not been obtained. This report does not contain any personal identifying information.

## Notation of prior abstract publication/presentation

None.

## Authorship

All authors attest that they meet the current ICMJE criteria for authorship.

## Authorship

All authors attest that they meet the current ICMJE criteria for Authorship.

## Funding

The authors declare that no funding or grant support was received for this study.

## Declaration of competing interest

The authors declare that they have no known competing financial interests or personal relationships that could have appeared to influence the work reported in this paper.
